# The Use of Bovine Teeth as an Experimental Substrate in the Evaluation of Shear in Contemporary Adhesive Systems

**DOI:** 10.3390/polym18151810

**Published:** 2026-07-24

**Authors:** Nazire Esra Ozer, Sahin Eren Akay, Ece Irem Oguz, Sadullah Uctaslı

**Affiliations:** 1 Department of Prothodontics, Faculty of Dentistry, Lokman Hekim University, 06530 Ankara, Turkey; esra.ozer@lokmanhekim.edu.tr (N.E.O.); erenakay38@gmail.com (S.E.A.); 2 Department of Prothodontics, Faculty of Dentistry, Ankara University, 06560 Ankara, Turkey; uctasli@dentistry.ankara.edu.tr

**Keywords:** shear bond strength, bovine teeth, human teeth, adhesive protocols

## Abstract

Evaluating the shear bond strength (SBS) of contemporary universal adhesive systems on different substrates is critical for understanding their clinical applicability. In this study, the SBS of universal adhesives was assessed on 144 human and bovine tooth specimens, comparing enamel and dentin across etch-and-rinse and self-etch protocols. Following single-step or two-step adhesive application and resin cementation, the specimens were subjected to 5000 thermocycles before being evaluated with a universal testing machine. Failure modes and surface morphology were additionally characterized using stereomicroscopy and scanning electron microscopy (SEM). Statistical analyses revealed that both tissue type (*p* = 0.043) and tooth type (*p* < 0.001) significantly influenced SBS, while the main effect of the adhesive protocol was not significant (*p* = 0.485). A significant interaction occurred between tissue type and adhesive protocol (*p* = 0.013); Post hoc analyses revealed that Group A enamel had significantly higher bond strength than both Group D enamel (*p* = 0.026) and Group A dentin (*p* = 0.001). Observed failures were predominantly adhesive or mixed, and SEM confirmed expected acid-induced surface roughness. Human teeth yielded higher bond strengths than bovine teeth, meaning absolute SBS values from bovine lab studies should be used with caution.

## 1. Introduction

Modern restorative dentistry aims to maximize both functional and aesthetic outcomes through minimally invasive approaches that prioritize the preservation of dental tissues, supported by continuously evolving adhesive technologies [1]. Achieving reliable bonding to dental substrates with distinct structural and chemical characteristics, such as enamel and dentin, remains a fundamental requirement for long-term clinical success [2]. Enamel, characterized by its highly mineralized and relatively homogeneous structure, differs markedly from dentin, which has a more complex composition, including organic components, and a tubular architecture [3]. These intrinsic differences significantly influence the interaction between adhesive systems and dental tissues, ultimately affecting bond strength and durability [1,2].

Adhesive technologies have evolved significantly over time to meet the need for improved bonding performance and simplified clinical procedures. Early adhesive systems required multiple application steps and were associated with technique sensitivity, particularly in etch-and-rinse approaches that involved separate acid etching, rinsing, priming, and bonding stages [4]. Subsequent developments led to the introduction of self-etch systems, which combined certain steps and eliminated the need for a separate rinsing phase, thereby reducing application time and minimizing operator-dependent variability [4,5]. More recently, universal adhesive systems have been developed to further enhance clinical versatility by allowing application in both etch-and-rinse and self-etch modes using a single formulation [6,7]. These systems are typically supplied in a single bottle containing all essential components, enabling simplified application protocols without compromising bonding effectiveness [7,8]. Such advancements reflect a continuous trend toward reducing procedural complexity while maintaining reliable adhesion across different substrates and clinical conditions.

To accurately evaluate the performance of adhesive systems, in vitro studies have traditionally relied on human teeth [2]. However, ethical concerns, biological variability, and challenges in specimen procurement have encouraged the use of alternative substrates [9]. Among these, bovine teeth are widely utilized due to their availability, absence of caries, and dimensions that facilitate standardized specimen preparation [10,11].

Comparative studies have demonstrated that bovine enamel and dentin share similar chemical characteristics with human dental tissues. In particular, no significant differences have been reported in calcium, phosphorus, and calcium-to-phosphorus ratios between human and bovine enamel [9]. Furthermore, investigations comparing various animal teeth have indicated that bovine dental tissues most closely resemble human teeth in terms of chemical composition [12], while elemental analyses have revealed a high degree of similarity between bovine and human hard tissues [13]. Despite these similarities, structural differences remain a critical consideration [9]. It has been reported that bovine dentin may present larger tubule diameters and variations in tubule density compared to human dentin, which could influence bonding behavior and limit the direct extrapolation of laboratory findings to clinical conditions [14,15]. As a result, the suitability of bovine teeth as a substitute for human teeth in bond-strength testing remains debated in the literature [9,15].

Although numerous studies have evaluated adhesive performance [9,12,13,15], investigations that simultaneously assess contemporary adhesive protocols across enamel and dentin in both human and bovine teeth remain limited. This gap highlights the need for further research to clarify the reliability of bovine teeth as an alternative substrate, particularly in the context of current universal adhesive systems.

The aim of this study was to comparatively evaluate the shear bond strength (SBS) of contemporary adhesive systems using bovine teeth as an experimental substrate, with respect to adhesive protocol, tissue type, and tooth type, and to investigate the potential interactions among these variables. Accordingly, four adhesive protocols were established by combining two universal adhesive systems (G-Premio Bond and G2-Bond Universal) with two application strategies (etch-and-rinse and self-etch). This design enabled the evaluation of adhesive performance under clinically relevant protocols while also allowing the assessment of the combined influence of adhesive formulation and surface conditioning strategy. Based on this experimental design, the following null hypotheses were proposed:

**H_01_.**  *Adhesive protocol has no statistically significant effect on SBS.*

**H_02_.**  *Tissue type has no statistically significant effect on SBS.*

**H_03_.**  *Tooth type has no statistically significant effect on SBS.*

**H_04_.**  *There is no statistically significant interaction among adhesive protocol, tissue type, and tooth type on bond strength.*

## 2. Materials and Methods

Considering the multifactorial nature of the experimental design, an a priori power analysis was conducted using G*Power 3.1.9.2 software (Heinrich Heine University Düsseldorf, Düsseldorf, Germany) to determine the minimum required sample size for a three-way factorial ANOVA model. The calculation was based on a medium standardized effect size (f = 0.30), as recommended by Cohen (1988) for factorial ANOVA designs [16], a significance level of α = 0.05, a statistical power of 80%, and numerator df = 3. Under these assumptions, the minimum required total sample size was calculated as 126 specimens [17,18]. To enhance the statistical robustness of the study and provide sufficient power for evaluating the multifactorial design, a total of 144 specimens (*n* = 9 per group) were included, exceeding the minimum requirement indicated by the power analysis.

Human permanent incisors extracted for periodontal reasons following approval from the Ethics Committee of Lokman Hekim University were collected (Approval No. 2026021). Bovine teeth were obtained from freshly extracted maxillasry incisors collected from a local slaughterhouse.

All selected teeth were cleaned of soft tissue remnants using periodontal curettes and stored in 0.5% chloramine-T solution at 4 °C for no longer than two months until testing [16]. The inclusion and exclusion criteria for the extracted teeth are summarized in Table 1 [9,17,18,19,20].

**Figure 1:**
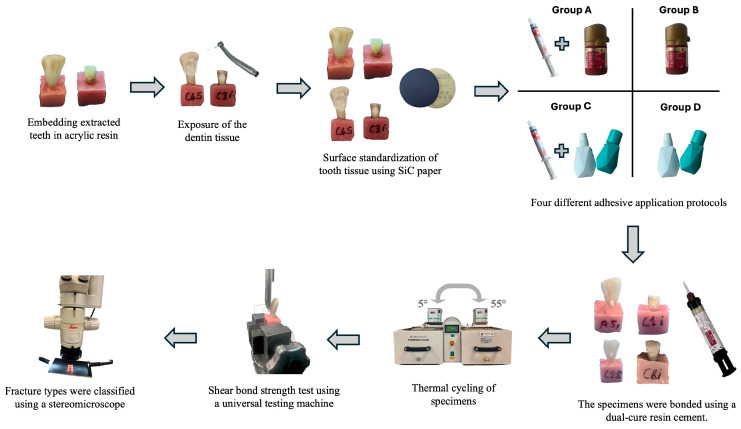
Schematic representation of the experimental design. Group A: etch-and-rinse + G-Premio Bond; Group B: G-Premio Bond (self-etch mode); Group C: etch-and-rinse + G2-Bond Universal; Group D: G2-Bond Universal (self-etch mode).

The overall study design is illustrated in Figure 1. The roots of the teeth were embedded in autopolymerizing acrylic resin (Meliodent, Kulzer, Germany), leaving the crowns exposed. During embedding, the teeth were positioned perpendicular to the mold base to ensure the bonding surface was aligned with the shear force direction during testing.

For the dentin groups, the labial enamel was removed using high-speed diamond burs under water cooling to expose mid-coronal dentin [7]. Subsequently, both enamel and dentin surfaces of human and bovine teeth were standardized by wet grinding with 600-grit silicon carbide (SiC) abrasive paper for 5 s [21,22].

The prepared enamel and dentin specimens (*N* = 144) were randomly allocated to 16 experimental groups (*n* = 9 per group) according to the adhesive protocol using a simple lottery method. Briefly, each specimen was assigned an identification number, and group allocation was performed by random drawing to minimize selection bias. All enamel and dentin surfaces were cleaned prior to bonding using a pumice-water slurry and a polishing rubber cup, followed by rinsing and air drying.

The adhesive protocols and materials used in this study are presented in Table 2 and Table 3. In Groups A and C, the etch-and-rinse protocol was applied using 37% phosphoric acid (i-Gel N; i-dental, Lithuania) for 15 s, followed by rinsing and gentle air drying. In Groups B and D, the self-etch mode was used without a separate acid-etching step.

During the adhesive application procedure, G-Premio Bond (GC, Tokyo, Japan) was used in Groups A and B. The adhesive was applied to the dry surface, left undisturbed for 10 s, and gently air dried for 5 s using an oil-free three-way syringe from a distance of approximately 10 cm to thoroughly evaporate the solvent and thin the adhesive into a homogenous, immobile film layer. It was then light cured for 10 s using an LED light curing unit (Celalux 2 High Power LED curing light; Voco GmbH, Cuxhaven, Germany) operating at a constant irradiance of 1000 mW/cm^2^, with the light guide tip positioned perpendicular and directly in contact with the specimen surface to ensure maximum polymerization efficacy. In Groups C and D, the G2-Bond Universal system (GC, Tokyo, Japan) was applied in a two-step procedure. First, the Universal Primer was applied to the surface and left for 10 s, followed by gentle air drying for 5 s under the same air stream criteria to secure adequate solvent evaporation. Subsequently, the Universal Bond was applied to achieve a homogenous film thickness and light cured for 10 s using the same LED curing unit positioned perpendicular and directly in contact with the surface (Table 3).

To evaluate surface morphology, an additional 24 specimens were prepared for scanning electron microscopy (SEM) analysis. The specimens included both human and bovine enamel and dentin samples, comprising untreated control specimens, etch-only specimens, and representative specimens from each experimental group following the application of different adhesive protocols. The specimens were mounted on aluminum stubs and sputter-coated with gold prior to examination. SEM analysis was performed using a scanning electron microscope (EVO 40; Carl Zeiss AG, Oberkochen, Germany). Surface morphology was evaluated at magnifications ranging from 1000× to 5000× to assess enamel prism structures and dentinal tubule morphology. For each specimen, multiple fields across the central surface area were examined. The micrographs presented in the manuscript were selected as representative images of the predominant morphological features consistently observed within each experimental group.

Following adhesive application, cylindrical polytetrafluoroethylene (PTFE) molds with an internal diameter of 3 mm and a height of 3 mm were positioned on the treated surfaces. A dual-cure resin cement (G-CEM LinkForce; GC, Tokyo, Japan) was applied into the molds according to the manufacturer’s instructions and light-cured for 20 s using the same LED curing unit operated at 1000 mW/cm^2^, with the light guide tip placed perpendicular and directly in contact with the top surface of the mold (0 mm distance) to ensure thorough polymer conversion throughout the 3 mm cement thickness.

Then, all specimens were subjected to thermocycling to simulate six months of intraoral aging. The specimens underwent 5000 cycles between 5 °C and 55 °C, with a dwell time of 30 s and a transfer time of 30 s between baths (Thermocycler 1100/1200; SD Mechatronik, Feldkirchen-Westerham, Germany).

Subsequently, the specimens were mounted in a universal testing machine (Model LRX-plus; Lloyd Instruments Ltd., Fareham, UK). A knife-edge loading device was applied at the cement–tooth interface in an inciso-gingival direction at a crosshead speed of 1 mm/min until failure. The maximum load at failure (N) was recorded, and SBS values were calculated in megapascals (MPa) using the following formula:

SBS = F_max_/A

where SBS is the shear bond strength, F_max_ is the maximum load at failure, and A is the bonded surface area. The bonding area was calculated using the formula A = πr^2^, corresponding to 7.07 mm^2^ for a specimen diameter of 3 mm.

To evaluate the failure mode, the fractured surfaces obtained after SBS testing were examined under a stereomicroscope at 20× magnification (Leica, Wetzlar, Germany). Failure modes were classified into three categories: adhesive, cohesive, and mixed. Adhesive failure was defined as the complete separation of the resin cement from the tooth surface with no residual cement remaining on the substrate. Cohesive failure was defined as failure occurring within the resin cement, enamel, or dentin. Mixed failure was characterized by the presence of both adhesive and cohesive failure patterns on the same fractured surface [23,24].

Statistical analyses were performed using IBM SPSS Statistics for Windows (Version 27.0; IBM Corp., Armonk, NY, USA). The normality of the data distribution was assessed using the Shapiro–Wilk test, and homogeneity of variances was evaluated using Levene’s test. For data meeting the assumptions of normality, a three-way factorial ANOVA was performed to evaluate the main effects of tooth type, tissue type, and adhesive protocol, as well as their interaction effects on bond strength. Post hoc comparisons were performed using the Bonferroni test. Statistical significance was set at *p* < 0.05.

## 3. Results

**Figure 2:**
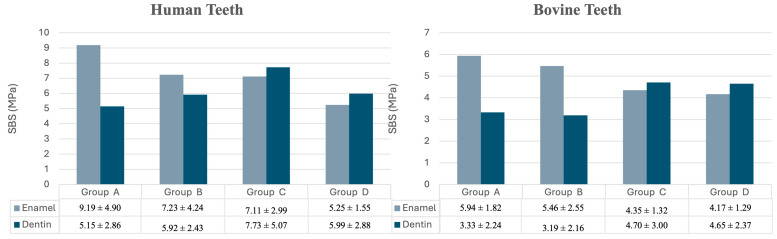
SBS values (MPa) across all experimental groups. Data are presented as mean ± standard deviation (SD). Group A: etch-and-rinse + G-Premio Bond; Group B: G-Premio Bond (self-etch mode); Group C: etch-and-rinse + G2-Bond Universal; Group D: G2-Bond Universal (self-etch mode).

The SBS values obtained after thermocycling are presented in Figure 2. In general, human teeth have shown higher bond strength values compared to bovine teeth. In Groups A and B, enamel surfaces exhibited higher bond strength values than dentin in both human and bovine teeth, whereas in Groups C and D, higher SBS values were observed in dentin compared to enamel. In human teeth, the highest mean enamel bond strength was recorded in Group A (9.19 ± 4.90 MPa), while the lowest value was observed in Group D (5.25 ± 1.55 MPa). For dentin, Group C exhibited the highest mean value (7.73 ± 5.07 MPa), whereas the lowest values were observed in Group A (5.15 ± 2.86 MPa). In bovine teeth, the highest mean enamel bond strength was also observed in Group A (5.94 ± 1.82 MPa), while the lowest value was recorded in Group D (4.17 ± 1.29 MPa). For dentin, Group C showed the highest mean bond strength (4.70 ± 3.00 MPa), whereas Group B showed the lowest (3.19 ± 2.16 MPa).

The results of the three-way ANOVA examining the effects of tissue type, adhesive protocol, and tooth type on bond strength are presented in Table 4.

The analysis revealed that both tissue type (enamel vs. dentin) and tooth type (human vs. bovine) had a statistically significant effect on bond strength (*p* = 0.043 and *p* < 0.001, respectively). In contrast, the adhesive protocol did not show a significant main effect (*p* = 0.485). A significant interaction between tissue type and adhesive protocol was identified (*p* = 0.013). No significant interactions were found between tooth type and the other variables (*p* > 0.05) (Table 4).

**Figure 3:**
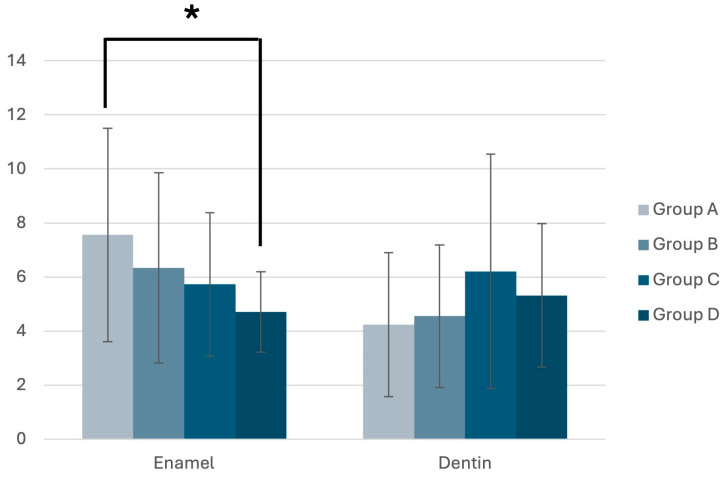
SBS values (MPa) according to adhesive protocol in enamel and dentin. Data are presented as mean ± standard deviation (SD). Group A: etch-and-rinse + G-Premio Bond; Group B: G-Premio Bond (self-etch mode); Group C: etch-and-rinse + G2-Bond Universal; Group D: G2-Bond Universal (self-etch mode). (*) indicates a statistically significant difference between Groups A and D in enamel (*p* < 0.05).

**Figure 4:**
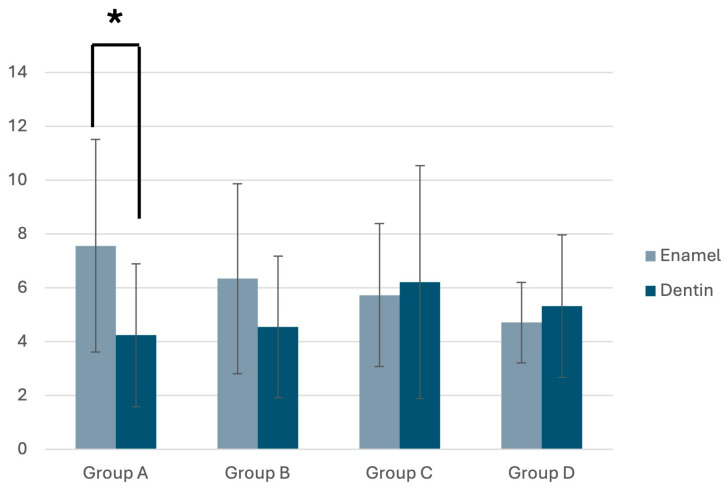
SBS values (MPa) of enamel and dentin according to different adhesive protocols. Data are presented as mean ± standard deviation (SD). Group A: etch-and-rinse + G-Premio Bond; Group B: G-Premio Bond (self-etch mode); Group C: etch-and-rinse + G2-Bond Universal; Group D: G2-Bond Universal (self-etch mode). (*) indicates a statistically significant difference between enamel and dentin within the same adhesive protocol (*p* < 0.05).

The detailed results of the interaction between tissue type and adhesive protocol are presented in Figure 3 and Figure 4. As no significant interaction between tooth type and the other variables was found (*p* > 0.05), the graphical representations were generated using the combined mean values of human and bovine data. For enamel, bond strength values in Group A were significantly higher than those in Group D (*p* = 0.026). In contrast, no statistically significant differences were observed among adhesive protocols in dentin (*p* > 0.05). Regarding tissue type, enamel exhibited significantly higher bond strength values than dentin within Group A (*p* = 0.001).

**Figure 5:**
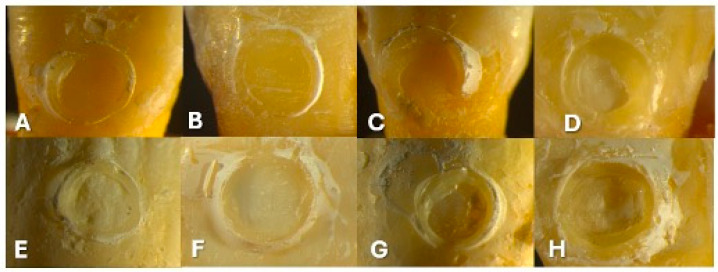
Representative stereomicroscopic images of failure modes after the SBS test. (**A**) Adhesive failure in human enamel; (**B**) adhesive failure in human dentin; (**C**) mixed failure in human enamel; (**D**) mixed failure in human dentin; (**E**) adhesive failure in bovine enamel; (**F**) adhesive failure in bovine dentin; (**G**) mixed failure in bovine enamel; (**H**) mixed failure in bovine dentin.

**Figure 6:**
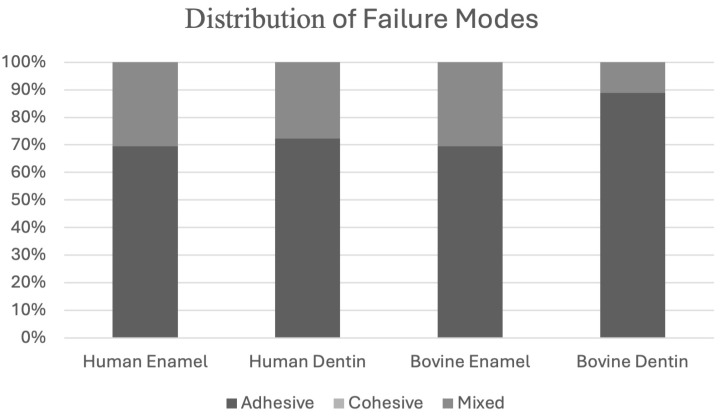
Distribution of failure modes after the SBS test.

Representative failure mode images and their distribution observed after the SBS test were evaluated under a stereomicroscope and are presented in Figure 5 and Figure 6. Analysis of failure patterns revealed that failures were predominantly adhesive and mixed across all groups, with no cohesive failures observed. In human enamel, 69.44% of failures were adhesive and 30.56% were mixed. In human dentin, the proportion of adhesive failures increased to 72.22%, whereas mixed failures accounted for 27.78%. A similar distribution was observed in bovine enamel, with 69.44% adhesive and 30.56% mixed failures. In contrast, bovine dentin showed a marked predominance of adhesive failures (88.89%), while mixed failures accounted for only 11.11%.

**Figure 7:**
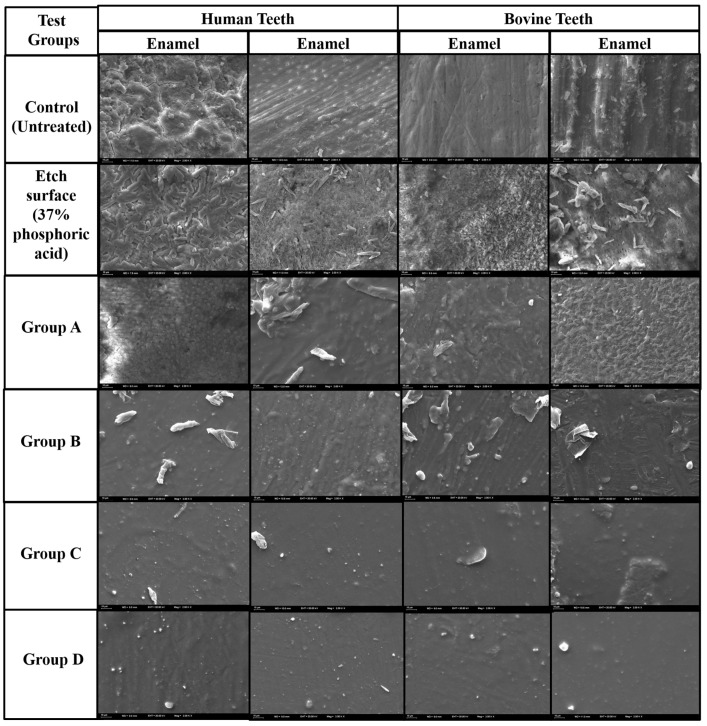
Representative SEM images showing enamel and dentin surface morphology under different treatment conditions. Group A: etch-and-rinse + G-Premio Bond; Group B: G-Premio Bond (self-etch mode); Group C: etch-and-rinse + G2-Bond Universal; Group D: G2-Bond Universal (self-etch mode). All images were obtained at a magnification of 2000×.

SEM analysis revealed surface morphological variations depending on the applied treatment. Representative images are presented in Figure 7. In untreated specimens, surface characteristics differed by tissue type. In enamel, prism structures appeared more clearly defined in human samples, whereas bovine enamel exhibited a comparatively less distinct prismatic pattern. In dentin, the surface showed an irregular, granular morphology, and tubule openings were not clearly distinguishable in either human or bovine specimens. Additionally, bovine dentin appeared more heterogeneous than human dentin.

Following phosphoric acid application, evident morphological alterations were observed in both enamel and dentin. Enamel surfaces in both human and bovine samples showed increased roughness with more apparent prismatic features and dissolution patterns. In dentin, the surface appeared more porous with visible tubule openings and increased irregularity; this effect was present in both substrates, with a tendency toward more pronounced irregularity in bovine dentin. In addition, localized granular or precipitate-like deposits were occasionally observed on both enamel and dentin surfaces.

In adhesive-treated groups (Groups A–D), both enamel and dentin surfaces exhibited a relatively uniform and continuous appearance. The underlying microstructural features, such as enamel prisms and dentinal tubule openings, were completely masked, confirming the presence of a continuous layer on the outermost surface. This resulted in a more homogeneous, film-like topography with a relatively smoother morphology. Topographical differences among adhesive protocols were subtle under surface SEM. However, minor localized irregularities were occasionally observed, particularly in Group A, where human enamel and bovine dentin showed slightly greater surface heterogeneity than in the other groups.

## 4. Discussion

The aim of this study was to evaluate the bonding performance of contemporary universal adhesives and to determine whether bovine teeth can serve as a suitable substitute for human teeth in dental research. Within this context, the effects of different application protocols (one- and two-step systems; etch-and-rinse and self-etch), tissue type (enamel and dentin), and tooth type (human and bovine) on bond strength were comparatively assessed.

Regarding the tested hypotheses, the adhesive protocol did not show a statistically significant main effect on bond strength. Therefore, H_01_ was accepted. In contrast, both tissue type and tooth type significantly influenced bond strength, leading to the rejection of H_02_ and H_03_. Specifically, enamel demonstrated higher bond strength values compared to dentin, and human teeth exhibited greater bond strength than bovine teeth. Furthermore, the significant interaction between adhesive protocol and tissue type led to the rejection of H_04_. These findings indicate that the adhesive protocol’s effect on bond strength depends on the type of dental tissue, as evidenced by the significant interaction between the two.

From a structural perspective, enamel and dentin exhibit fundamentally different bonding behaviors [25,26]. Enamel, due to its highly mineralized structure and low organic content, provides a relatively stable bonding substrate [26,27]. Following phosphoric acid etching, well-defined microretentive patterns are formed by exposing the enamel prism ends, facilitating effective resin infiltration and enhancing micromechanical interlocking [4,28]. In the present study, phosphoric acid etching was applied in Groups A and C, whereas Groups B and D were treated without prior etching. Accordingly, the relatively higher bond strength values observed in enamel, particularly in Group A compared with Group D, may be associated with both its intrinsic structural characteristics and the effect of surface conditioning. This is further supported by the observation that enamel exhibited higher bond strength than dentin under the same protocol, highlighting the more favorable bonding conditions it provides.

These interpretations are supported by SEM findings, showing increased surface roughness and more pronounced prismatic features in enamel following phosphoric acid application, consistent with classical etching patterns that promote micromechanical retention. In this context, the higher bond strength values observed with the etch-and-rinse approach compared with the self-etch mode in enamel suggest that universal adhesives may be limited in their ability to produce sufficient micromechanical retention when used without prior acid conditioning. The relatively higher pH of universal adhesives compared with phosphoric acid may result in a more superficial demineralization pattern, thereby restricting effective resin penetration and reducing bond strength [29].

In contrast, dentin presents a more complex and less predictable bonding substrate due to its higher organic content, tubular structure, and intrinsic moisture [26,30]. These characteristics increase susceptibility to hydrolytic degradation, especially following thermocycling, and may compromise the stability of the adhesive interface [30,31]. Thermal stresses have been reported to increase the risk of microleakage and interfacial degradation at the dentin adhesive interface [32,33]. SEM observations in the present study further revealed a more porous and irregular dentin surface following phosphoric acid application, indicating surface demineralization and open dentinal tubules. While these structural alterations modify the substrate topography to prepare it for subsequent micromechanical interlocking [28], the inherently heterogeneous nature of dentin may also introduce greater morphological variability. In addition, the occasional presence of granular or precipitate-like deposits on both enamel and dentin surfaces may be associated with dissolution and reprecipitation processes following acid application, as previously described for calcium phosphate phases formed under acidic conditions [34]. These findings suggest that both microstructural and potential chemical alterations on the outermost surface may influence the initial conditioning of dental tissues prior to adhesive application.

Consistent with these observations, previous studies have shown that universal adhesives tend to provide higher bond strength in enamel when applied with the etch-and-rinse approach, and selective enamel etching has therefore been recommended to enhance micromechanical retention [35,36]. However, other studies have reported comparable performance between etch-and-rinse and self-etch modes, suggesting that the effectiveness of the application strategy may depend on the specific adhesive system used [37,38].

Further emphasizing the technique sensitivity of substrate conditioning, a study by Campos et al. (2020) demonstrated that prior phosphoric acid etching significantly decreased the bond strength of several single-step universal adhesives to dentin substrates [39]. This finding supports the notion that, unlike enamel, dentin may be adversely affected by aggressive pre-etching when using certain universal adhesive formulations, likely due to over-demineralization and incomplete resin infiltration into the exposed collagen network [39]. In contrast, a recent study by Zhao et al. (2025) evaluating a multi-step resin cement protocol reported that omission of either the phosphoric acid etch or the tooth primer step significantly weakened bonding to enamel, highlighting that enamel adhesion remains highly dependent on effective surface conditioning and primer interaction to prevent interfacial gaps and ensure long-term stability [40].

In line with the present results, these findings suggest that selective etching improves enamel bonding, whereas in dentin, the choice of protocol appears to depend more on the adhesive system’s characteristics and formulation than on routine phosphoric acid etching.

When evaluated by tissue type, no consistent trend was observed across all adhesive protocols. In Groups A and B, where the one-step system (G-Premio Bond) was applied, enamel exhibited higher bond strength values, whereas in Groups C and D, where the two-step system (G2-Bond Universal) was used, dentin demonstrated bond strength values comparable to or higher than those of enamel. This pattern indicates that the influence of tissue type on bond strength is not uniform and varies with the adhesive protocol.

One-step universal adhesives combine hydrophilic and hydrophobic components within a single application step, making them more sensitive to substrate conditions, particularly moisture control [26,41]. The presence of water and solvents in these simplified systems has been associated with phase separation and reduced polymerization efficiency, potentially compromising bonding performance in dentin [42,43]. On the other hand, the relatively improved dentin bonding observed with the two-step adhesive system may be attributed to its application strategy and material composition. The incorporation of a separate priming stage may enhance resin infiltration into the moist dentin matrix and improve interaction with the exposed collagen network [44,45]. Previous studies have shown that the use of a separate primer can facilitate more effective monomer penetration into the demineralized dentin matrix and contribute to more stable hybrid layer formation [26,45].

In line with these considerations, the relatively higher bond strength observed in enamel for Group A may be due to the combined effects of phosphoric acid etching and the adhesive composition, which together enhance micromechanical retention at the enamel surface. Conversely, in dentin, the improved performance observed with the two-step system suggests that simplified application alone may not be sufficient to achieve optimal bonding in more complex substrates. Supporting this, Daneshpooy et al. (2025) reported that two-bottle universal adhesives may perform better than one-step systems in dentin under etch-and-rinse conditions, while showing comparable performance in self-etch mode [46].

Beyond differences in application strategy, the present findings suggest that the chemical formulation of universal adhesive systems may also contribute to the substrate-dependent bonding behavior observed in this study. Contemporary universal adhesives are methacrylate-based polymer systems composed of functional acidic monomers, cross-linking dimethacrylates, solvents, water, and photoinitiator systems, whose bonding effectiveness depends on both chemical interaction with hydroxyapatite and the formation of a stable polymerized adhesive layer after light curing [47,48]. In the present study, the one-step adhesive system (G-Premio Bond) showed relatively higher bond strength in enamel, whereas the two-step adhesive system (G2-Bond Universal) demonstrated comparable or higher bond strength in dentin. Although adhesive protocol was not identified as an independent determinant of bond strength, this substrate-dependent behavior may reflect differences in adhesive polymer formulation. Unlike G-Premio Bond, which incorporates functional monomers, solvents, and polymerizable resin components within a single formulation, G2-Bond Universal separates the hydrophilic primer from a HEMA-free hydrophobic bonding resin. Such formulation strategies have been proposed to influence monomer infiltration, solvent evaporation, and adhesive layer formation according to the physicochemical characteristics of enamel and dentin [48,49,50]. Therefore, the present findings indicate that bonding performance should be interpreted not only according to the clinical application strategy but also according to the interaction between adhesive polymer formulation and substrate characteristics. Although polymerization kinetics and interfacial chemistry were not directly evaluated, these formulation-related differences may partly explain the substrate-dependent bonding behavior observed in the present study.

SEM observations further supported these findings by demonstrating that adhesive treated surfaces in all groups exhibited a relatively uniform, continuous, and film-like morphology, in which underlying microstructural features such as enamel prisms and dentinal tubule openings were no longer clearly distinguishable. This finding suggests effective surface coverage by the adhesive systems and is consistent with previous reports describing the ability of universal adhesives to form continuous interfacial layers on dental substrates [51]. Minor localized irregularities were occasionally observed, particularly in Group A, which may be associated with variations in adhesive viscosity and solvent content. In contrast, the more homogeneous and smoother film appearance observed in the two-step system groups reflects improved wettability. This is consistent with recent findings by Nagasawa et al. (2025), who demonstrated that the G2-Bond Universal system maintains its immediate bond strength and adaptable spreading capacity even when air-thinned to a reduced film thickness [52].

These observations are consistent with previous reports indicating that adhesive film homogeneity and interfacial continuity play a critical role in bonding performance and marginal integrity [45,53,54,55]. Although the masking of dentinal tubule openings may be consistent with adhesive penetration into the conditioned substrate, the presence and quality of the hybrid layer cannot be confirmed from surface SEM images alone and would require additional ultrastructural analyses. Therefore, rather than indicating a definitive superiority of one system over another, the present findings suggest that bonding performance is influenced by both substrate characteristics and the specific application conditions, and that clinical outcomes may depend on appropriate protocol selection and technique sensitivity.

In addition to the effects of adhesive protocol and tissue type, tooth type also played a significant role in bonding performance. Although bovine teeth have been widely proposed as an alternative to human teeth in adhesive research [14,56], the consistently higher bond strength values observed in human specimens in the present study indicate that bovine teeth may not fully reproduce the absolute bonding performance of human substrates. These differences can be explained by underlying microstructural variations between the two substrates. Bovine enamel has been reported to exhibit a less regular prismatic organization [57], which may affect the formation and uniformity of etching-induced microporosities and consequently influence adhesive penetration [26,58]. Likewise, interspecies differences in dentin tubule characteristics, including diameter and distribution, may alter the interaction between adhesive systems and the substrate [9]. The presence of larger tubules, together with increased fluid content, may negatively affect the mechanical stability of the hybrid layer [59,60].

However, recent evidence suggests that these interspecies differences may be influenced by the adhesive protocol used. For example, Oltramare et al. (2026) showed that while human dentin outperforms bovine dentin in etch-and-rinse adhesive systems, the performance of both substrates was comparable when self-etch adhesives were applied, indicating that the relative difference in bond strength may be protocol-dependent [61]. Similarly, other studies comparing human and bovine enamel reported minimal differences in enamel bond strength when a selective etching strategy was employed, despite microstructural distinctions [62,63]. These findings imply that while absolute bond strength values may differ between substrates, the comparative trends across experimental conditions remain consistent, supporting the validity of bovine teeth in comparative adhesive research.

SEM observations in the present study were consistent with these findings. Human enamel showed more clearly defined prism structures, whereas bovine enamel exhibited a less distinct prismatic pattern. In dentin, both substrates presented a generally irregular and granular surface morphology without clearly distinguishable tubule openings, although bovine dentin appeared more heterogeneous. Such differences may contribute to the variability observed in bond strength values between the two substrates. Despite these structural differences, both human and bovine enamel and dentin exhibited comparable morphological responses to surface treatments, suggesting that the fundamental mechanisms governing adhesion are similar across the two substrates. In agreement with previous reports, this supports the use of bovine teeth as a suitable experimental model, particularly when evaluating comparative trends rather than absolute values [64].

However, this comparability primarily applies to relative trends across experimental conditions rather than to the magnitude of bond strength values. Therefore, while bovine teeth provide a practical and standardized substrate for in vitro studies, caution should be exercised when directly extrapolating bond strength values to clinical conditions.

The SBS values observed in the present study may appear lower than those reported in some previous investigations evaluating universal adhesives [65,66]. However, direct comparisons between studies should be made with caution because bond strength values are highly sensitive to specimen preparation, bonded surface area, aging protocols, and substrate characteristics. In the present study, all specimens were subjected to 5000 thermocycles before testing. This rigorous artificial aging procedure is known to induce hydrolytic degradation and thermal stress which naturally reduces bond strength at the adhesive interface [65,66]. Furthermore, the predominance of adhesive failures observed across all groups suggests that the measured values accurately reflected the actual integrity of the adhesive interface rather than cohesive failures within the substrate. Therefore, despite the relatively low absolute values, the findings indicate that both human and bovine substrates are capable of maintaining a functional and stable adhesive bond under challenging conditions. Ultimately, the study was designed primarily to compare the behavior of human and bovine substrates under strictly standardized conditions rather than to maximize absolute bond strength values.

The potential influence of donor age and periodontal status on bonding behavior should be considered when interpreting the present findings. Age-related changes in dentin, including secondary dentin deposition, dentinal tubule narrowing, increased mineralization, and dentinal sclerosis, may alter substrate permeability and affect adhesive interaction with dental tissues [67]. Previous studies have reported that older dentin may exhibit lower bond strength values than younger dentin because of these structural modifications [67,68]. In addition, because the human teeth were obtained from donors requiring extraction for periodontal reasons, some degree of biological variability among specimens cannot be excluded [68]. Although standardized preparation and storage procedures were applied to all specimens, individual differences in tissue characteristics, mineralization patterns, and pre-existing structural alterations may have contributed to substrate heterogeneity. These factors are difficult to control in studies using extracted teeth and represent an inherent limitation of laboratory bond strength investigations [67,68]. Therefore, the results should be interpreted with consideration of the natural variability that exists among dental substrates.

To further interpret the bonding performance, failure mode analysis was evaluated in relation to the bond strength findings. The predominance of adhesive and mixed failures, along with the absence of cohesive failures, indicates that failure mainly occurred at the resin-tooth interface, reflecting interfacial integrity. The presence of mixed failures, particularly in enamel groups, may indicate partial retention of resin on the substrate, suggesting that bonding was not uniformly weak and that stress distribution at the interface was, to some extent, effective. In contrast, the higher proportion of adhesive failures observed in dentin, especially in bovine specimens, reflects a more interface-dominated failure pattern, supporting the notion that dentin bonding is more sensitive and less predictable than enamel bonding.

When comparing tooth types, the similar distribution of failure modes in enamel between human and bovine specimens supports the comparable bonding behavior observed in these substrates. However, the marked predominance of adhesive failures in bovine dentin may be associated with its structural characteristics, such as wider dentinal tubules, higher tubule density, and differences in the mineral–organic matrix composition [64,69]. These features may negatively influence hybrid layer stability and contribute to weaker interfacial adhesion. Overall, the failure mode patterns observed in the present study are consistent with the bond strength results, reinforcing that enamel provides a more stable and reliable bonding substrate, whereas dentin, particularly bovine dentin, exhibits greater susceptibility to interfacial failure.

Several in vitro methods, including SBS, microtensile (μTBS), microshear (μSBS), and interfacial fracture toughness tests, are available for evaluating adhesive performance [70].In the present study, the SBS test was selected because it is a simple, reproducible, and widely accepted method for comparing adhesive systems under standardized laboratory conditions [70,71].However, it should be recognized that the knife-edge loading configuration may produce non-uniform stress concentrations at the adhesive interface, which can influence the measured bond strength values [71]. Although μTBS, μSBS, and fracture toughness tests may provide a more detailed assessment of interfacial behavior, they require more complex specimen preparation and are generally more technique-sensitive [72].Therefore, the SBS test was considered appropriate for the comparative design of the present study, while its inherent methodological limitations should be taken into account when interpreting the findings.

This study has several limitations. The thermocycling protocol applied (5000 cycles) corresponds to approximately six months of clinical aging [65,66], and therefore the findings should be interpreted in terms of short-term bonding performance. Furthermore, while thermal stress provides a baseline aging effect, thermocycling alone cannot fully replicate the complex multifactorial clinical degradation mechanisms of the oral cavity, which include continuous moisture exposure, intraoral pH variations, enzymatic activity, and mechanical fatigue under masticatory loads. The in vitro design, the use of a shear bond strength test, and the evaluation of a single resin cement further limit the direct clinical extrapolation of the results.

Future studies should incorporate extended aging protocols, including longer thermocycling periods and additional methods to better assess the hydrolytic stability of the adhesive interface. The inclusion of different dentin depths, the use of complementary mechanical testing approaches, such as microtensile bond strength, microshear bond strength, and interfacial fracture toughness tests, together with comparisons involving multiple resin cements and adhesive systems, as well as statistical analyses of failure mode distributions, would provide a more comprehensive understanding of adhesive performance and the relationship between bond strength and fracture behavior.

## 5. Conclusions

Within the limitations of this study, bond strength was significantly influenced by both the dental tissue and the tooth type, indicating that adhesive performance depends on how each substrate interacts with the selected protocol. Although SEM observations demonstrated broadly similar surface morphological patterns after conditioning, bovine teeth yielded significantly lower bond strength values than human teeth. These findings suggest that bonding effectiveness is governed not only by surface topography but also by intrinsic structural and compositional differences between substrates. Therefore, bovine teeth may serve as a suitable substrate for standardized comparative laboratory studies and for evaluating relative differences between adhesive systems when consistent experimental conditions are maintained. However, because absolute bond strength values differed significantly from those obtained with human teeth, bovine teeth should not be considered a direct substitute for human substrates when extrapolating in vitro findings to clinical performance.

## Figures and Tables

**Table 1:** Inclusion and exclusion criteria for specimen selection.

Tooth Type	Inclusion Criteria	Exclusion Criteria
Human Teeth	Extracted for periodontal reasons Intact, caries-free buccal/labial enamel surface with no cracks or fractures caused during extraction Sufficient crown size for testing	Presence of any restoration (e.g., filling, crown) Developmental anomalies such as enamel hypoplasia or amelogenesis imperfecta
Bovine Teeth	Intact labial enamel surface Anatomical structure allowing preparation of a wide and flat surface	Presence of macroscopic cracks or fractures on the crown surface Surface damage during extraction or transportation Severe structural defects or excessive wear

**Table 2:** Chemical composition of the materials used in the study.

Material	Chemical Composition	Manufacturer
G-CEM LinkForce	Paste A: Bis-GMA, UDMA, DMA, initiator Paste B: Bis-MEPP, UDMA, DMA, initiator, Bis-EMA, dibenzoyl peroxide, BHT Filler: Barium glass	GC, Tokyo, Japan
G-Premio Bond	MDP, MDTP, 4-MET, methacrylate monomer, acetone, silica	GC, Tokyo, Japan
G2-Bond Universal	Primer: 4-MET, MDP, MDTP, dimethacrylate monomer, acetone, water, photoinitiator, filler Adhesive: Dimethacrylate monomer, Bis-GMA, filler, photoinitiator	GC, Tokyo, Japan
i-GEL N	37% phosphoric acid	i-dental, Lithuania

(Bis-GMA: bisphenol A glycidyl methacrylate; UDMA: urethane dimethacrylate; DMA: dimethacrylate; Bis-MEPP: bisphenol A ethoxylated dimethacrylate phosphate; Bis-EMA: ethoxylated bisphenol A dimethacrylate; BHT: butylated hydroxytoluene; MDP: 10-methacryloyloxydecyl dihydrogen phosphate; MDTP: methacryloyloxydecyl dihydrogen thiophosphate; 4-MET: 4-methacryloxyethyl trimellitate anhydride).

**Table 3:** Adhesive Application Protocols.

Test Groups	Tooth Type	Tissue Type
		Enamel/Dentin
Group A	Human/Bovine	37% phosphoric acid applied for 15 s, rinsed for 5 s, and air-dried. G-Premio Bond applied for 10 s, gently air-dried for 5 s, and light-cured for 10 s.
Group B	Human/Bovine	G-Premio Bond applied for 10 s, gently air-dried for 5 s, and light-cured for 10 s.
Group C	Human/Bovine	37% phosphoric acid applied for 15 s, rinsed for 5 s, and air-dried. G2-Bond Universal Primer applied for 10 s, air-dried for 5 s, followed by G2-Bond Universal Bond and light-cured for 10 s.
Group D	Human/Bovine	G2-Bond Universal Primer applied for 10 s, air-dried for 5 s, followed by G2-Bond Universal Bond and light-cured for 10 s.

**Table 4:** Three-way ANOVA results for the effects of tissue type (enamel and dentin), adhesive protocol (Groups A-D), and tooth type (human and bovine) on bond strength.

Source	Type III Sum of Squares	df	Mean Square	F	*p*	Effect Size (Partial η^2^)
Corrected Model	353.922	15	23.595	2.717	0.001 *	0.242
Intercept	4490.870	1	4490.870	517.127	<0.001 *	0.802
Tissue Type	36.320	1	36.320	4.182	0.043 *	0.032
Adhesive Protocol	21.371	3	7.124	0.820	0.485	0.019
Tooth Type	177.569	1	177.569	20.447	<0.001 *	0.138
Tissue Type × Adhesive Protocol	97.397	3	32.466	3.738	0.013 *	0.081
Tissue Type × Tooth Type	0.002	1	0.002	0.000	0.987	0.000
Adhesive Protocol × Tooth Type	14.212	3	4.737	0.545	0.652	0.013
Tissue Type × Adhesive Protocol × Tooth Type	7.051	3	2.350	0.271	0.846	0.006
Error	1111.585	128	8.684			
Total	5956.378	144				
Corrected Total	1465.508	143				

Type III sums of squares were used. Effect sizes are reported as partial eta squared (η^2^p). Statistical significance was set at * *p* < 0.05. (df: degrees of freedom; F: F statistic).

## Data Availability

The data supporting the findings of this study are available from the corresponding author upon reasonable request.

## Abbreviations

The following abbreviations are used in this manuscript:

SBS	Shear bond strength
SEM	Scanning electron microscopy/Scanning electron microscope
ANOVA	Analysis of variance
SiC	Silicon carbide
LED	Light-emitting diode
PTFE	Polytetrafluoroethylene
MPa	Megapascals
SPSS	Statistical Package for the Social Sciences
SD	Standard deviation
μTBS	Microtensile
μSBS	Microshear

## References

[1] Evli N.N., Kocaaydın S. (2025). Dentin Bağlayıcı Ajanlarda Güncel Gelişmeler. J. Int. Dent. Sci..

[2] De Munck J., Van Landuyt K., Peumans M., Poitevin A., Lambrechts P., Braem M., Van Meerbeek B. (2005). A critical review of the durability of adhesion to tooth tissue: Methods and results. J. Dent. Res..

[3] Kazak M., Dönmez N. (2019). Development of Dentin Bonding Systems from Past to Present. Bezmialem Sci..

[4] Sofan E., Sofan A., Palaia G., Tenore G., Romeo U., Migliau G. (2017). Classification review of dental adhesive systems: From the IV generation to the universal type. Ann. Stomatol..

[5] Muñoz M.A., Luque-Martinez I., Malaquias P., Hass V., Reis A., Campanha N.H., Loguercio A.D. (2015). In Vitro Longevity of Bonding Properties of Universal Adhesives to Dentin. Oper. Dent..

[6] Hong X., Huang Z., Tong Z., Jiang H., Su M. (2021). Clinical effects of different etching modes for universal adhesives: A systematic review and meta-analysis. Ann. Palliat. Med..

[7] Bourgi R., Kharouf N., Cuevas-Suárez C.E., Lukomska-Szymanska M., Haikel Y., Hardan L. (2024). A Literature Review of Adhesive Systems in Dentistry: Key Components and Their Clinical Applications. Appl. Sci..

[8] Muñoz M.A., Luque I., Hass V., Reis A., Loguercio A.D., Bombarda N.H.C. (2013). Immediate bonding properties of universal adhesives to dentine. J. Dent..

[9] Al Ankily M., Baz S., Mahmoud H., Bakr M.M., Shamel M. (2025). Bovine Teeth as Substitutes for Human Teeth in Dental Research: Ultrastructural and Radiographic Analysis. Eur. J. Dent..

[10] Franchini Pan Martinez L., Ferraz N.K.L., Lannes A.C.N.L., Rodrigues M.C., De Carvalho M.F., Zina L.G., Suzuki T.Y.U., Moreira A.N., Magalhães C.S. (2023). Can bovine tooth replace human tooth in laboratory studies? A systematic review. J. Adhes. Sci. Technol..

[11] Hong J.-Y., Kang E.-J., Kwon J.-S. (2025). Whitening efficacy and enamel properties of 30% hydrogen peroxide solution incorporated with strontium-containing Fluorapatite. Sci. Rep..

[12] Teruel J.D.D., Alcolea A., Hernández A., Ruiz A.J.O. (2015). Comparison of chemical composition of enamel and dentine in human, bovine, porcine and ovine teeth. Arch. Oral Biol..

[13] Möhring S., Cieplik F., Hiller K.A., Ebensberger H., Ferstl G., Hermens J., Zaparty M., Witzgall R., Mansfeld U., Buchalla W. (2023). Elemental Compositions of Enamel or Dentin in Human and Bovine Teeth Differ from Murine Teeth. Materials.

[14] de Carvalho M.F.F., Leijôto-Lannes A.C.N., Rodrigues M.C.N., Nogueira L.C., Ferraz N.K.L., Moreira A.N., Yamauti M., Zina L.G., Magalhães C.S. (2018). Viability of Bovine Teeth as a Substrate in Bond Strength Tests: A Systematic Review and Meta-analysis. J. Adhes. Dent..

[15] Sahebi S., Sobhnamayan F., Hasani S., Mahmoodi N., Dadgar D. (2024). Bovine and Ovine Teeth as a Substitute for the Human Teeth: An Experimental Study. J. Dent..

[16] Cohen J. (1988). Statistical Power Analysis for the Behavioral Sciences.

[17] Limas J., Mauricio F., Alvitez-Temoche D., Mauricio-Vilchez C., Medina J., Mayta-Tovalino F. (2020). Effect of Post-bleaching Surface Microroughness with Whiteness HP Blue vs Whiteness HP Maxx on Different Locations of Bovine Enamel. J. Contemp. Dent. Pract..

[18] Gaile M., Papia E., Zalite V., Locs J., Soboleva U. (2022). Resin Cement Residue Removal Techniques: In Vitro Analysis of Marginal Defects and Discoloration Intensity Using Micro-CT and Stereomicroscopy. Dent. J..

[19] Fu Y., Ekambaram M., Li K.C., Zhang Y., Cooper P.R., Mei M.L. (2024). In Vitro Models Used in Cariology Mineralisation Research—A Review of the Literature. Dent. J..

[20] Goyal S., Virk R., Bhullar K.K., Sahiwal H., Kant D., Deepanshu D. (2025). Tooth Substrate vs Conventional Materials Used as a Base in Class V Cavities: A Comparative Study. Int. J. Clin. Pediatr. Dent..

[21] Ando S., Watanabe T., Tsubota K., Yoshida T., Irokawa A., Takamizawa T., Kurokawa H., Miyazaki M. (2008). Effect of adhesive application methods on bond strength to bovine enamel. J. Oral Sci..

[22] Reis A.F., Giannini M., Kavaguchi A., Soares C.J., Line S.R. (2004). Comparison of microtensile bond strength to enamel and dentin of human, bovine, and porcine teeth. J. Adhes. Dent..

[23] Ismatullaev A., Taşın S., Usumez A. (2021). Evaluation of bond strength of resin cement to Er:YAG laser-etched enamel and dentin after cementation of ceramic discs. Lasers Med. Sci..

[24] Vongtavatchai V., Niyatiwatchanchai B., Srinivasan M., Porntaveetus T., Tagami J., Srijunbarl A., Siripamitdul K., Nantanapiboon D. (2025). Effects of Different Dentin Surface Cleaning Protocols on Bond Strength of Dual-Cure Resin Cement Following Temporary Cementation. Eur. J. Dent..

[25] Alkattan R. (2023). Adhesion to enamel and dentine: An update. Prim. Dent. J..

[26] Perdigão J. (2020). Current perspectives on dental adhesion: (1) Dentin adhesion—Not there yet. Jpn. Dent. Sci. Rev..

[27] Belmar da Costa M., Delgado A.H.S., Pinheiro de Melo T., Amorim T., Mano Azul A. (2021). Analysis of laboratory adhesion studies in eroded enamel and dentin: A scoping review. Biomater. Investig. Dent..

[28] Saikaew P., Sattabanasuk V., Harnirattisai C., Chowdhury A.F.M.A., Carvalho R., Sano H. (2022). Role of the smear layer in adhesive dentistry and the clinical applications to improve bonding performance. Jpn. Dent. Sci. Rev..

[29] Iwase K., Takamizawa T., Sai K., Shibasaki S., Barkmeier W.W., Latta M.A., Kamimoto A., Miyazaki M. (2022). Early Phase Enamel Bond Performance of a Two-step Adhesive Containing a Primer Derived from a Universal Adhesive. J. Adhes. Dent..

[30] Mokeem L.S., Garcia I.M., Melo M.A. (2023). Degradation and Failure Phenomena at the Dentin Bonding Interface. Biomedicines.

[31] Betancourt D.E., Baldion P.A., Castellanos J.E. (2019). Resin-Dentin Bonding Interface: Mechanisms of Degradation and Strategies for Stabilization of the Hybrid Layer. Int. J. Biomater..

[32] Malysa A., Wezgowiec J., Grzebieluch W., Danel D.P., Wieckiewicz M. (2022). Effect of Thermocycling on the Bond Strength of Self-Adhesive Resin Cements Used for Luting CAD/CAM Ceramics to Human Dentin. Int. J. Mol. Sci..

[33] Breschi L., Mazzoni A., Ruggeri A., Cadenaro M., Di Lenarda R., De Stefano Dorigo E. (2008). Dental adhesion review: Aging and stability of the bonded interface. Dent. Mater..

[34] Feitosa V.P., Bazzocchi M.G., Putignano A., Orsini G., Luzi A.L., Sinhoreti M.A., Watson T.F., Sauro S. (2013). Dicalcium phosphate (CaHPO4·2H2O) precipitation through ortho- or meta-phosphoric acid-etching: Effects on the durability and nanoleakage/ultra-morphology of resin-dentine interfaces. J. Dent..

[35] Rosa W.L., Piva E., Silva A.F. (2015). Bond strength of universal adhesives: A systematic review and meta-analysis. J. Dent..

[36] Wong J., Tsujimoto A., Fischer N.G., Baruth A.G., Barkmeier W.W., Johnson E.A., Samuel S.M., Takamizawa T., Latta M.A., Miyazaki M. (2020). Enamel Etching for Universal Adhesives: Examination of Enamel Etching Protocols for Optimization of Bonding Effectiveness. Oper. Dent..

[37] Chen H., Feng S., Jin Y., Hou Y., Zhu S. (2022). Comparison of bond strength of universal adhesives using different etching modes: A systematic review and meta-analysis. Dent. Mater. J..

[38] Perdigão J. (2010). Dentin bonding-variables related to the clinical situation and the substrate treatment. Dent. Mater..

[39] Campos M., Moura D.M.D., Borges B.C.D., Assuncao I.V., Caldas M., Platt J.A., Özcan M., Souza R. (2020). Influence of Acid Etching and Universal Adhesives on the Bond Strength to Dentin. Braz. Dent. J..

[40] Zhao M., Sato T., Khaled A.H.M., Ikeda M., Shimada Y. (2025). The influence of phosphoric acid and primer treatment on the evaluation of the adhesive resin cement/enamel interface. Dent. Mater. J..

[41] Nagarkar S., Theis-Mahon N., Perdigão J. (2019). Universal dental adhesives: Current status, laboratory testing, and clinical performance. J. Biomed. Mater. Res. Part B Appl. Biomater..

[42] Van Landuyt K.L., Snauwaert J., De Munck J., Peumans M., Yoshida Y., Poitevin A., Coutinho E., Suzuki K., Lambrechts P., Van Meerbeek B. (2007). Systematic review of the chemical composition of contemporary dental adhesives. Biomaterials.

[43] Yoshida Y., Yoshihara K., Hayakawa S., Nagaoka N., Okihara T., Matsumoto T., Minagi S., Osaka A., Van Landuyt K., Van Meerbeek B. (2012). HEMA inhibits interfacial nano-layering of the functional monomer MDP. J. Dent. Res..

[44] Pashley D.H., Tay F.R., Breschi L., Tjäderhane L., Carvalho R.M., Carrilho M., Tezvergil-Mutluay A. (2011). State of the art etch-and-rinse adhesives. Dent. Mater..

[45] Van Meerbeek B., Yoshihara K., Yoshida Y., Mine A., De Munck J., Van Landuyt K.L. (2011). State of the art of self-etch adhesives. Dent. Mater..

[46] Daneshpooy M., Bahari M., Dabaghi Tabriz F., Alizadeh-Oskoee P., Fahim Z. (2025). Effect of Accelerated Aging on the Microtensile Bond Strength of a Two-Step Adhesive Containing a Universal Adhesive Primer, an in vitro study. Open Dent. J..

[47] Tang C., Ahmed M.H., Yao C., Mercelis B., Yoshihara K., Peumans M., Van Meerbeek B. (2023). Bonding performance of experimental HEMA-free two-step universal adhesives to low C-factor flat dentin. Dent. Mater..

[48] Delgado A.H.S., Ahmed M.H., Nunes Ferreira M., Mano Azul A., Polido M., Yoshihara K., Van Meerbeek B. (2025). Physico-Chemical Properties and Performance of Functional Monomers Used in Contemporary Dental Adhesive Technology. J. Adhes. Dent..

[49] Tsujimoto A., Fischer N.G., Barkmeier W.W., Latta M.A. (2022). Bond Durability of Two-Step HEMA-Free Universal Adhesive. J. Funct. Biomater..

[50] Özlü A., Tonga G., Hatırlı H. (2026). Evaluation of microtensile bond strength to dentin and adhesive layer thickness of a new generation two-bottle universal adhesive. Eur. Oral Res..

[51] Santos V.L., Medina A.N.T., Barros A.P.O., Escalante-Otárola W.G., de Melo Alencar C., Kuga M.C. (2026). Effects of desensitizing treatments on acid-etched dentin: Debris precipitation, bond strength and tag formation. Odontology.

[52] Nagasawa Y., Eda Y., Matsumoto S., Nakajima H., Hibino Y. (2025). Effect of air-thinning a two-step adhesive layer on bond strength to tooth substrates. Odontology.

[53] Hanabusa M., Mine A., Kuboki T., Momoi Y., Van Ende A., Van Meerbeek B., De Munck J. (2012). Bonding effectiveness of a new ‘multi-mode’ adhesive to enamel and dentine. J. Dent..

[54] Cuevas-Suárez C.E., da Rosa W.L.O., Lund R.G., da Silva A.F., Piva E. (2019). Bonding Performance of Universal Adhesives: An Updated Systematic Review and Meta-Analysis. J. Adhes. Dent..

[55] Triani F., Pereira da Silva L., Ferreira Lemos B., Domingues J., Teixeira L., Manarte-Monteiro P. (2022). Universal Adhesives: Evaluation of the Relationship between Bond Strength and Application Strategies—A Systematic Review and Meta-Analyses. Coatings.

[56] Soares F.Z., Follak A., da Rosa L.S., Montagner A.F., Lenzi T.L., Rocha R.O. (2016). Bovine tooth is a substitute for human tooth on bond strength studies: A systematic review and meta-analysis of in vitro studies. Dent. Mater..

[57] Olek A., Klimek L., Bołtacz-Rzepkowska E. (2020). Comparative scanning electron microscope analysis of the enamel of permanent human, bovine and porcine teeth. J. Vet. Sci..

[58] Wang C., Li Y., Wang X., Zhang L., Tiantang, Fu B. (2012). The enamel microstructures of bovine mandibular incisors. Anat. Rec..

[59] Pegado R.E., do Amaral F.L., Flório F.M., Basting R.T. (2010). Effect of different bonding strategies on adhesion to deep and superficial permanent dentin. Eur. J. Dent..

[60] Liu Y., Tjäderhane L., Breschi L., Mazzoni A., Li N., Mao J., Pashley D.H., Tay F.R. (2011). Limitations in bonding to dentin and experimental strategies to prevent bond degradation. J. Dent. Res..

[61] Oltramare R., Lutz Guzman C.A., Lotz J.J., Attin T., Wegehaupt F.J. (2026). Bovine Dentin as a Substitute for Human Dentin: Bond Strength Tests on Sound and Eroded Substrate. Dent. J..

[62] Arslandaş Dinçtürk B., Kedici Alp C. (2025). Evaluation of the Effect of Different Universal Adhesives on Remineralized Enamel by Shear Bond Strength and Fe-SEM/EDX Analysis. J. Funct. Biomater..

[63] Sato T., Takagaki T., Hatayama T., Nikaido T., Tagami J. (2021). Update on Enamel Bonding Strategies. Front. Dent. Med..

[64] Yassen G.H., Platt J.A., Hara A.T. (2011). Bovine teeth as substitute for human teeth in dental research: A review of literature. J. Oral Sci..

[65] Çerçi Akçay H. (2025). Evaluation of In-Vitro Aging Procedures in Dentistry: A Traditional Review. Dent. Med. J.-Rev..

[66] Gale M.S., Darvell B.W. (1999). Thermal cycling procedures for laboratory testing of dental restorations. J. Dent..

[67] Giannini M., Chaves P., Oliveira M.T. (2003). Effect of tooth age on bond strength to dentin. J. Appl. Oral Sci..

[68] Marshall G.W., Marshall S.J., Kinney J.H., Balooch M. (1997). The dentin substrate: Structure and properties related to bonding. J. Dent..

[69] Camargo C.H., Siviero M., Camargo S.E., de Oliveira S.H., Carvalho C.A., Valera M.C. (2007). Topographical, diametral, and quantitative analysis of dentin tubules in the root canals of human and bovine teeth. J. Endod..

[70] Armstrong S., Breschi L., Özcan M., Pfefferkorn F., Ferrari M., Van Meerbeek B. (2017). Academy of Dental Materials guidance on in vitro testing of dental composite bonding effectiveness to dentin/enamel using micro-tensile bond strength (μTBS) approach. Dent. Mater..

[71] Braga R.R., Meira J.B., Boaro L.C., Xavier T.A. (2010). Adhesion to tooth structure: A critical review of “macro” test methods. Dent. Mater..

[72] Van Meerbeek B., De Munck J., Yoshida Y., Inoue S., Vargas M., Vijay P., Van Landuyt K., Lambrechts P., Vanherle G. (2003). Buonocore memorial lecture. Adhesion to enamel and dentin: Current status and future challenges. Oper. Dent..

